# Uterine Rupture by Intended Mode of Delivery in the UK: A National Case-Control Study

**DOI:** 10.1371/journal.pmed.1001184

**Published:** 2012-03-13

**Authors:** Kathryn E. Fitzpatrick, Jennifer J. Kurinczuk, Zarko Alfirevic, Patsy Spark, Peter Brocklehurst, Marian Knight

**Affiliations:** 1 National Perinatal Epidemiology Unit, University of Oxford, Oxford, United Kingdom; 2 Division of Perinatal and Reproductive Medicine, University of Liverpool, Liverpool Women's Hospital, Liverpool, United Kingdom; University of Queensland, Australia

## Abstract

**Background:**

Recent reports of the risk of morbidity due to uterine rupture are thought to have contributed in some countries to a decrease in the number of women attempting a vaginal birth after caesarean section. The aims of this study were to estimate the incidence of true uterine rupture in the UK and to investigate and quantify the associated risk factors and outcomes, on the basis of intended mode of delivery.

**Methods and Findings:**

A UK national case-control study was undertaken between April 2009 and April 2010. The participants comprised 159 women with uterine rupture and 448 control women with a previous caesarean delivery. The estimated incidence of uterine rupture was 0.2 per 1,000 maternities overall; 2.1 and 0.3 per 1,000 maternities in women with a previous caesarean delivery planning vaginal or elective caesarean delivery, respectively. Amongst women with a previous caesarean delivery, odds of rupture were also increased in women who had ≥ two previous caesarean deliveries (adjusted odds ratio [aOR] 3.02, 95% CI 1.16–7.85) and <12 months since their last caesarean delivery (aOR 3.12, 95% CI 1.62–6.02). A higher risk of rupture with labour induction and oxytocin use was apparent (aOR 3.92, 95% CI 1.00–15.33). Two women with uterine rupture died (case fatality 1.3%, 95% CI 0.2–4.5%). There were 18 perinatal deaths associated with uterine rupture among 145 infants (perinatal mortality 124 per 1,000 total births, 95% CI 75–189).

**Conclusions:**

Although uterine rupture is associated with significant mortality and morbidity, even amongst women with a previous caesarean section planning a vaginal delivery, it is a rare occurrence. For women with a previous caesarean section, risk of uterine rupture increases with number of previous caesarean deliveries, a short interval since the last caesarean section, and labour induction and/or augmentation. These factors should be considered when counselling and managing the labour of women with a previous caesarean section.

*Please see later in the article for the Editors' Summary*

## Abstract 2

A case-control study using UK data estimates the risk of uterine rupture in subsequent deliveries amongst women who have had a previous caesarean section.

## Editors' Summary

**Background:**

Uterine rupture is a serious complication of pregnancy in which the wall of the uterus (womb) tears during pregnancy or early labor. Signs and symptoms of uterine rupture include fetal heart rate abnormalities, abdominal pain, and vaginal bleeding. If uterine rupture happens during labor, the woman must have an immediate caesarean section (surgical delivery of her baby) to save both her life and that of her baby. The woman's womb and nearby organs can be damaged at rupture or removed during surgery and she may need a blood transfusion because of severe bleeding. Moreover, her baby may develop respiratory distress syndrome and other life-threatening complications. In high income countries, uterine rupture most commonly occurs in women who have delivered a previous pregnancy by caesarean section. In a caesarean section, the baby is delivered through a cut made through the abdominal wall and the uterine wall. The stretching that occurs during pregnancy or the strong contractions of labor can tear the scar left by this cut, resulting in uterine rupture.

**Why Was This Study Done?:**

Women who have had a caesarean delivery are generally encouraged to try to deliver subsequent babies vaginally. However, recent reports of an increased risk of complications (morbidity) and death (mortality) due to uterine rupture are thought to reduce women's willingness to attempt vaginal birth after caesarean (VBAC) in some countries. In the UK, for example, where one in four babies is delivered by caesarean section, a previous caesarean delivery is one of commonest reasons for a repeat section. Obstetricians (doctors who care for women during child birth) need to know as much as possible about the incidence of uterine rupture and about the risk factors for it so that they can advise women who have had a previous caesarean section about their delivery options. In this national case-control study (a study that compares the characteristics of people with and without a specific condition), the researchers estimate the incidence of uterine rupture in the UK by intended mode of delivery and investigate and quantify the risk factors for and outcomes of uterine rupture.

**What Did the Researchers Do and Find?:**

The researchers used the UK Obstetric Surveillance System (UKOSS) to identify all the women in the UK who had a uterine rupture over a 13-month period (159 women, 139 of whom had had a previous caesarean delivery). Controls for the study were women who had not had a uterine rupture but who had previously delivered by caesarean section. Overall, the incidence of uterine rupture was 0.2 per 1,000 maternities. In women with a previous caesarean delivery, 2.1 and 0.3 per 1,000 maternities ended in uterine rupture in women planning vaginal delivery and caesarean delivery, respectively. Amongst women who had had a previous caesarean delivery, the risk of uterine rupture was greater among those who had had two or more previous caesarean deliveries or a caesarean delivery less than 12 months previously, or whose labor was induced. Two women died following uterine rupture (a case fatality of 1.3%) and 18 babies died around the time of birth (a perinatal mortality rate of 124 per 1,000 live births; the UK perinatal mortality rate is 7.5 per 1,000 live births). 15 of the women who had a uterine rupture had their womb removed, 10 had other organs damaged, and nearly half had other complications; 19 of the surviving babies had health problems.

**What Do These Findings Mean?:**

These findings indicate that, in the UK, although uterine rupture is associated with significant mortality and morbidity, it is a rare occurrence even among women who have had a previous caesarean delivery and are planning a vaginal delivery. They also indicate that, for women who have previously had a caesarean section, the risk of rupture increases with the number of previous caesarean deliveries, with a short interval since the last caesarean section, and with labor induction. Although the researchers may not have identified all the women who had a uterine rupture during the study period or may have identified only the worst cases, these findings provide valuable information about the factors that obstetricians need to consider when advising women who have previously had a caesarean section and when managing their labor.

**Additional Information:**

Please access these Web sites via the online version of this summary at http://dx.doi.org/10.1371/journal.pmed.1001184.

- This study is linked to a *PLoS Medicine* Research Article by Caroline Crowther and a *PLoS Medicine* Perspective by Catherine Spong
- Wikipedia has a page on uterine rupture (note: Wikipedia is a free online encyclopedia that anyone can edit; available in several languages)
- The American Congress of Obstetricians and Gynecologists has information sheets for patients on caesarean sections and on vaginal birth after caesarean delivery
- The Royal College of Obstetricians and Gynaecologists in the UK has information for women on birth after previous caesarean
- Childbirth Connection, a US-based not-for-profit organization, provides information about caesarean sections and about vaginal birth after caesarean
- The National Childbirth Trust, a UK charity, provides information for parents on all aspects of pregnancy and birth, including caesarean sections and vaginal birth after caesarean delivery
- The UK charity Healthtalkonline has personal stories from women making decisions about birth after a caesarean section
- A personal story of uterine rupture during an attempted VBAC is available
- The UK Obstetric Surveillance System studies rare disorders of pregnancy in the UK

## Introduction

Uterine rupture is a complication of pregnancy associated with severe maternal and fetal morbidity and mortality. In high income countries it most commonly occurs in women who have previously delivered by caesarean section [1]. This observation has led to debate about the optimal management of labour and delivery in women who have delivered by caesarean section in previous pregnancies. Women with a previous caesarean delivery have generally been encouraged to attempt a trial of labour in subsequent pregnancies [2], but recent reports of an increased risk of morbidity, particularly due to uterine rupture, are thought to have contributed to a marked decrease in some countries in the number of women attempting vaginal birth after caesarean section [3]. Indeed, the rate of caesarean section delivery in the UK is increasing, with previous caesarean section being the most common primary obstetric indication for repeat section [4].

Three systematic reviews have identified a number of deficiencies with the existing studies of uterine rupture in high income countries, hampering the accurate assessment of the incidence and risk factors for this complication [1],[5],[6]. One of the reviews suggested that a multicentre prospective cohort study or national registry would offer the best opportunity to inform preventive strategies [5]. The aim of this study, therefore, was to carry out a national population-based case-control study using the UK Obstetric Surveillance System (UKOSS) to estimate the incidence of uterine rupture in the UK and to investigate and quantify the associated risk factors and outcomes.

## Methods

### Ethics Statement

This study was approved by the London Research Ethics Committee (ref 09/H0718/8).

### Study Power

A national population-based case-control study was undertaken. Over the 13-mo study period, we anticipated identifying 200 cases (on the basis of an estimated incidence of 1 in 4,000 maternities [7]) and 600 controls. A ratio of three controls per case was planned in the study proposal to maximise the power of the study, given that uterine rupture is a rare condition and the number of cases would be limited by disease incidence. Assuming 10% of women with a previous caesarean section delivering in the UK are induced with prostaglandin and/or receive oxytocin in their labour, and with a 3 to 1 ratio of controls to cases, 106 cases and 316 controls would give an estimated power of 80% at the 5% level of statistical significance to detect a 2.5-fold increase in the odds of uterine rupture in women with a previous caesarean section who have prostaglandin labour induction and/or oxytocin used in labour.

### Case Definition

Cases were all women in the UK identified as having a uterine rupture defined as a complete separation of the wall of the pregnant uterus, with or without expulsion of the fetus, involving rupture of membranes at the site of the uterine rupture or extension of the complete separation of the wall of the uterus into uterine muscle separate from any previous scar, and endangering the life of the mother or fetus. Any asymptomatic palpable or visualised defect, noted incidentally at caesarean delivery, for example, was excluded.

### Control Definition

Controls were defined as any woman delivering a fetus or infant who had not suffered from a uterine rupture and who had delivered by caesarean section in any previous pregnancy regardless of the mode of delivery of the current pregnancy.

### Data Collection

Cases were identified through the monthly mailing of the UKOSS [8] between 1st April 2009 and 30th April 2010. Nominated clinicians in each obstetrician-led maternity unit in the UK were sent a card each month and asked to report all cases of uterine rupture, thus covering the entire cohort of UK births in the study period. Clinicians who reported a case were then asked to complete a data collection form for the case, detailing demographic and other potential risk factors, management, and outcomes. Previous studies using this methodology have suggested good case ascertainment [9],[10]. Controls were obtained from a random sample of obstetrician-led maternity units in the UK in month 4 and month 12 of the study, weighted by the total number of births. The time and day on which reporting clinicians were asked to select controls were randomly identified using data on birth date and time from one region of the UK (Leicestershire), to try and provide a representative sample of women delivering during each 24-h period and on different days of the week. Clinicians were asked to complete a data collection form for the controls that was identical to those used for cases with the exception of the details of the uterine rupture. Up to five reminders were sent if completed forms were not returned. All data requested were anonymous. On receipt of data collection forms, cases were checked to confirm that they met the case definition. Duplicate reports were identified by comparing the woman's year of birth and expected date of delivery.

### Statistical Analysis

The overall incidence with 95% CIs of uterine rupture was calculated using the most recently available national birth data (2009 and 2010 for England and Wales [11] and 2009 for Scotland [12] and Northern Ireland [13]) as a proxy denominator for the number of maternities during the study period. To calculate the incidence with 95% CIs of uterine rupture in women with and without a previous caesarean section, the most recently available birth data were used together with an estimate of the proportion of women in the UK who had previously delivered by caesarean section (15%), derived from the rate in a group of population-based controls comprised of women giving birth in the UK in 2005–2006 [14]. This group of population-based controls were identified as the two women delivering immediately before a woman who had a peripartum hysterectomy in the UK during the period from February 2005 to February 2006, and are comparable in characteristic to the available national data on women giving birth in the UK. Information on the proportion of women with a previous caesarean delivery planning a vaginal or caesarean section delivery in their current pregnancy, estimated from that observed in the control women, was used to estimate the denominator for calculation of the incidence and 95% CI of uterine rupture according to planned mode of delivery in women with a previous caesarean section. Denominator data to allow calculation of the incidence and 95% CI of uterine rupture in women with a prior caesarean delivery planning a vaginal delivery according to whether labour was induced with or without prostaglandin and/or oxytocin were also estimated using the proportions observed in the control women.

Potential risk factors for uterine rupture after prior delivery by caesarean section were evaluated by comparing the women with a previous caesarean delivery who had a uterine rupture to the control group of women. Odds ratios (ORs) with 95% CIs were estimated using unconditional logistic regression. A full regression model was developed by including both explanatory and potential confounding factors in a core model if there was a preexisting hypothesis or evidence to suggest they were causally related to uterine rupture, for example, number of previous caesarean section deliveries. Factors with a high proportion of missing data (>20%) were omitted from the full model where there was no evidence (*p*>0.20) in the unvariate analysis that they were associated with uterine rupture. Continuous variables were tested for departure from linearity by the addition of first-order fractional polynomials to the model and subsequent likelihood ratio testing. Where there was evidence for nonlinearity, continuous variables were presented and treated as categorical in the analysis. Where there was no evidence of departure from linearity, continuous variables are presented as categorical for ease of interpretation, but have been treated as continuous linear terms when adjusting for them in the analysis. Plausible interactions were tested in the full regression model by the addition of interaction terms and subsequent likelihood ratio testing on removal, with a *p*-value of 0.01 considered evidence of significant interaction to account for multiple testing. Unconditional logistic regression was also used to compare the sociodemographic, parity, previous uterine surgery, and infant birthweight characteristics of women who had a uterine rupture in the absence of a previous caesarean delivery to the group of population-based controls comprised of women giving birth in the UK in 2005–2006 [14]. The Wilcoxon rank-sum test was used to compare medians and Fisher's exact test was used to compare proportions where appropriate. All analyses were carried out using STATA v11 software.

## Results

**Figure 1:**
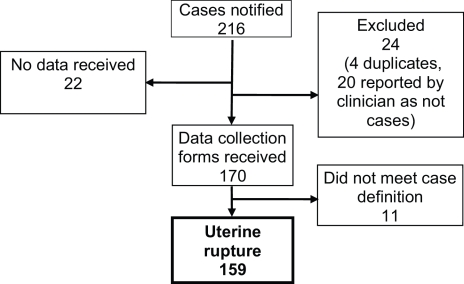
Case reporting and completeness of data collection.

All 223 UK hospitals with obstetrician-led maternity units contributed data to UKOSS during the study period, representing 100% participation. Of the 216 notified cases of uterine rupture, data collection was complete for 90% (Figure 1). There were 159 confirmed cases of uterine rupture in an estimated 852,206 maternities [11]–[13], representing an estimated incidence of 1.9 per 10,000 maternities (95% CI 1.6–2.2). Table 1 shows the estimated incidence of uterine rupture in different categories of women. Data collection forms were received for 448 controls (75% of those requested).

**Table 1:** Estimated incidence of uterine rupture in different categories of women.

Category			Number of Women with a Uterine Rupture	Estimated Number of Maternities	Estimated Incidence of Uterine Rupture (95% CI) per 10,000 Maternities
Women without a previous caesarean delivery			20	724,375	0.3 (0.2–0.4)
Women with a previous caesarean delivery			139	127,831	11 (9–13)
Women with a previous caesarean delivery planning:					
	Elective caesarean delivery in current pregnancy		20	71,585	3 (2–4)
	Vaginal delivery in current pregnancy		116	56,246	21 (17–25)
Women with a previous caesarean delivery planning a vaginal delivery in current pregnancy and:					
	Laboured without prostaglandin induction^a^ or oxytocin used in labour		52	41,622	13 (9–16)
	Labour induced with prostaglandins and/or oxytocin used in labour		44	14,624	30 (22–40)
		Labour induced with prostaglandin and oxytocin not used in labour	10	2,812	36 (17–65)
		Laboured without prostaglandin induction^a^ but oxytocin used in labour	28	10,124	28 (18–40)
		Labour induced with prostaglandin and oxytocin used in labour	6	1,687	36 (13–77)

a Labour either not induced or induced without prostaglandin.

### Presentation of Uterine Rupture

**Figure 2:**
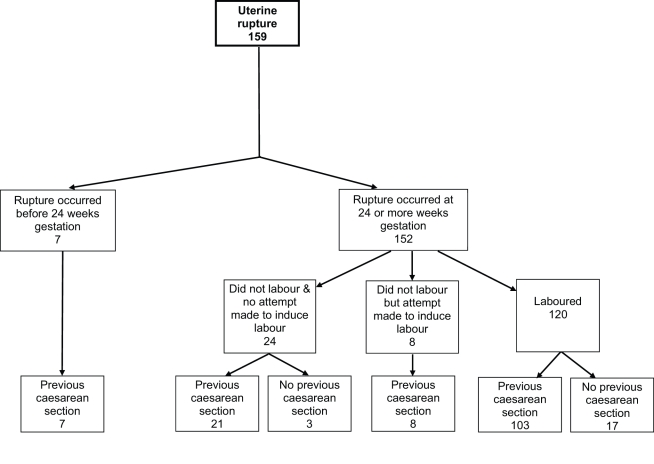
Uterine rupture cases by gestational age at rupture, labour, and previous caesarean section status.

The median gestational age at diagnosis of uterine rupture was 39 wk (range 8–42 wk) (Figure 2). All seven of the women who had their rupture diagnosed before 24 wk gestation had a previous delivery by caesarean section and five occurred in association with medical termination of pregnancy. Of the 152 ruptures diagnosed at ≥24 wk gestation, the majority (120/152, 79%) occurred in women who laboured, the median time between diagnosis of labour and diagnosis of rupture being 6.6 h. 21 ruptures diagnosed at or after 24 wk gestation occurred in women with a previous caesarean section who did not labour or have an attempt made at inducing their labour (Figure 2). Compared to the control women, these women were more likely to have placenta praevia (14% versus 1% in control women, unadjusted OR [uOR] 24.72, 95% CI 4.66–131.10). No other significant differences were found, but note the limited power of this analysis due to small numbers.

Table 2 shows the clinical symptoms or signs noted prior to the diagnosis of rupture. Fetal heart rate abnormality was the commonest symptom noted, affecting the fetuses of 118 of the women (76%). Bradycardia was the most frequent abnormality, occurring in the fetuses of 40 women. 97 women (62%) presented with a combination of symptoms, the most frequent being abdominal pain and fetal heart rate abnormality, occurring in 28 women.

**Table 2:** Symptoms and signs noted prior to diagnosis of uterine rupture.

Symptoms and Signs	*n* (%)^a^ Cases (*n* = 159)
Fetal heart rate abnormality	118 (76)
Abdominal pain	76 (49)
Vaginal bleeding	45 (29)
Altered uterine contractions	21 (13)
Hypotension/fainting/cardiac arrest	10 (6)
Haematuria	4 (3)
Other^b^	21 (13)

a Percentage of individuals with complete data.

b Includes shoulder tip pain, scar tenderness, maternal tachycardia, and blood in abdomen.

The 21 women with a previous caesarean delivery who had their rupture diagnosed at or after 24 wk gestation in the absence of labour or attempt at inducing labour, presented as follows: 15 presented with abdominal pain and/or vaginal bleeding (eight before and seven at or after 37 wk), with a fetal heart rate abnormality noted in eight of these; three had rupture before 37 wk in association with placenta praevia; one presented at 37 wk with maternal collapse; and two had fetal heart rate abnormalities noted following membrane rupture (one before and one at 37 wk).

### Risk Factors for Uterine Rupture after Prior Delivery by Caesarean Section

**Figure 3:**
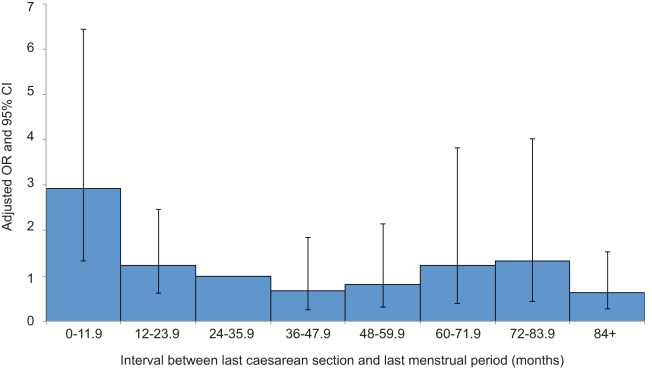
Risk of uterine rupture according to the interval between the last caesarean section and start of current pregnancy. Adjusted for woman's age as a continuous linear term, ethnicity, body mass index as a continuous linear term, parity as a continuous linear term, number of previous caesarean deliveries as a continuous linear term, previous uterine surgery, placenta praevia, macrosomia, and planned mode of delivery.

A total of 139 (87%) of the uterine ruptures occurred in women who had previously delivered by caesarean section. Table 3 shows the characteristics of these women compared to control women. Women who had two or more previous caesarean deliveries had a raised odds of having a uterine rupture compared to women with only one previous caesarean delivery (adjusted OR [aOR] 3.02, 95% CI 1.16–7.85), as did women who had an interval of less than 12 mo compared to ≥24 mo between their last caesarean section and their last menstrual period in their current pregnancy (aOR 3.12, 95% CI 1.62–6.02). There was no evidence to suggest a departure from linearity in the relationship between odds of rupture and number of caesarean deliveries, with the odds of rupture increasing by 3.02 (95% CI 1.62–5.63) for every one additional caesarean delivery. However, there was evidence of a departure in linearity in the association between uterine rupture and caesarean section-pregnancy interval, with the odds of rupture appearing to plateau for intervals beyond 12 mo (Figure 3).

**Table 3:** Risk factors for uterine rupture in women with prior delivery by caesarean section.

Risk Factor	*n* (%)^a^ Cases with a Previous Caesarean (*n = *139)	*n* (%)^a^ Controls (*n = *448)	uOR (95% CI)	*p*-Value	aOR (95% CI)^b^	*p* -Value
Sociodemographic factors						
**Age (y)**						
Less than 35	94 (68)	313 (70)	1	0.5922	1	0.1347
35 or older	45 (32)	134 (30)	1.12 (0.74–1.68)		1.47 (0.89–2.45)	
**Ethnic group**						
White	94 (69)	325 (75)	1		1	
Non-white	42 (31)	111 (25)	1.31 (0.86–2.00)	0.213	1.12 (0.68–1.84)	0.6611
**Socio-economic group**						
Managerial and professional occupations	33 (30)	108 (32)	1			
Other	77 (70)	226 (68)	1.12 (0.70–1.78)	0.6482		
**Body mass index at booking (kg/m^2^)**						
Less than 25	56 (42)	173 (40)	1		1	
25–29.9	43 (33)	132 (31)	1.01 (0.64–1.59)	0.9783	1.12 (0.65–1.91)	0.6871
30 or more	33 (25)	127 (29)	0.8 (0.49–1.31)	0.3768	0.73 (0.41–1.30)	0.2852
Previous obstetric and medical history						
**Parity**						
1–2	116 (83)	385 (86)	1		1	
3 or more	23 (17)	62 (14)	1.23 (0.73–2.07)	0.4344	1.1 (0.57–2.14)	0.7767
**Number of previous caesarean deliveries**						
1	121 (87)	368 (82)	1		1	
2 or more	18 (13)	79 (18)	0.69 (0.40–1.20)	0.1925	3.02 (1.16–7.85)	0.0232
**Previous caesarean uterine incision type(s)**						
All low transverse incisions	120 (99)	390 (98)	1			
Any non-low transverse incisions	1 (1)	8 (2)	0.41 (0.05–3.28)	0.398		
**Previous caesarean uterine closure type(s)**						
All double	75 (90)	241 (91)	1			
All single	5 (6)	17 (6)	0.95 (0.34–2.65)	0.9145		
Mixture of double and single or other closure type	3 (4)	7 (3)	1.38 (0.35–5.46)	0.6488		
**Previous uterine surgery**						
No	124 (90)	394 (88)	1		1	
Yes	14 (10)	52 (12)	0.86 (0.46–1.60)	0.6237	0.92 (0.43–1.96)	0.8325
**Previous uterine perforation**						
No	137 (100)	446 (100)				
Yes	0 (0)	1 (0)				
Current pregnancy						
**Twin pregnancy**						
No	139 (100)	444 (99)				
Yes	0 (0)	4 (1)				
**Interval between last caesarean section and last menstrual period (mo)**						
24 or more	71 (52)	294 (67)	1		1	
12–23	35 (26)	99 (22)	1.46 (0.92–2.33)	0.1078	1.38 (0.80–2.38)	0.2488
Less than 12	31 (23)	48 (11)	2.67 (1.59–4.50)	0.0002	3.12 (1.62–6.02)	0.0007
**Placenta praevia**						
No	136 (98)	445 (99)	1		1	
Yes	3 (2)	3 (1)	3.27 (0.65–16.40)	0.1494	28.19 (4.03–197.39)	0.0008
**Macrosomia (birthweight 4,000 g or more)**						
No	117 (89)	382 (86)	1		1	
Yes	14 (11)	62 (14)	0.74 (0.40–1.36)	0.332	0.85 (0.42–1.73)	0.6614
**Planned mode of delivery**						
Elective caesarean section	20 (15)	250 (56)	1		1	
Vaginal	116 (85)	198 (44)	7.32 (4.40–12.19)	<0.0001	19.37 (8.53–43.98)	<0.0001

a Percentage of individuals with complete data.

b Adjusted for all factors in the table apart from socio-economic group, previous uterine incision type(s), previous caesarean uterine closure type(s), previous uterine perforation, and twin pregnancy. When adjusting for age, body mass index, parity, and number of previous caesarean deliveries, these variables have been treated as a continuous linear term in the analysis.

The presence of placenta praevia also increased the odds of rupture (aOR 28.19, 95% CI 4.03–197.39), although note that this finding is based on a very small number of women and should be interpreted with caution. The odds of rupture was also raised in women who planned to have a vaginal delivery in their current pregnancy compared to women who planned to deliver by elective caesarean section (aOR 19.37, 95% CI 8.53–43.98). This finding was irrespective of whether the women who planned to have a vaginal delivery had their labour induced and/or received oxytocin in labour (Table 4). However, the women who had prostaglandin labour induction and/or oxytocin used in labour appeared to have raised odds of rupture compared to the women who laboured without prostaglandin induction or oxytocin in labour (Table 5). No significant interactions were found.

**Table 4:** Risk factors for uterine rupture in women with prior delivery by caesarean section.

Risk Factor	*n* (%)^a^ Cases with a Previous Caesarean (*n = *139)	*n* (%)^a^ Controls (*n = *448)	uOR (95% CI)	*p*-Value	aOR (95% CI)^b^	*p*-Value
Planned elective caesarean section delivery	20 (17)	250 (58)	1		1	
Planned vaginal delivery and:						
Laboured without prostaglandin induction^c^ or oxytocin in labour	52 (45)	132 (31)	4.92 (2.82–8.60)	<0.0001	12.74 (5.44–29.87)	<0.0001
Labour induced with prostaglandin and oxytocin not used in labour	10 (9)	9 (2)	13.89 (5.06–38.10)	<0.0001	35.91 (10.38–124.28)	<0.0001
Laboured without prostaglandin induction^c^ but oxytocin in labour	28 (24)	33 (8)	10.61 (5.38–20.91)	<0.0001	35.36 (13.38–93.41)	<0.0001
Labour induced with prostaglandin and oxytocin used in labour	6 (5)	5 (1)	15 (4.21–53.48)	<0.0001	52.05 (11.30–239.84)	<0.0001

a Percentage of individuals with complete data.

b Adjusted for woman's age as a continuous linear term, ethnicity, body mass index as a continuous linear term, parity as a continuous linear term, number of previous caesarean deliveries as a continuous linear term, previous uterine surgery, interval between last caesarean section and last menstrual period as a categorical term, placenta praevia, and macrosomia.

c Labour either not induced or induced without prostaglandin.

**Table 5:** Risk factors for uterine rupture in women with prior delivery by caesarean section who planned to have a vaginal delivery in current pregnancy.

Risk Factor	*n* (%)^a^ Cases with a Previous Caesarean Who Planned a Vaginal Delivery (*n = *116)	*n* (%)^a^ Controls Who Planned a Vaginal Delivery (*n = *198)	uOR (95% CI)	*p*-Value	aOR (95% CI)^b^	*p*-Value
Laboured without prostaglandin induction^c^ or oxytocin in labour	52 (54)	132 (74)	1		1	
Labour induced with prostaglandin and oxytocin not used in labour	10 (10)	9 (5)	2.82 (1.08–7.34)	0.0335	2.66 (0.93–7.63)	0.0677
Laboured without prostaglandin induction^c^ but oxytocin in labour	28 (29)	33 (18)	2.15 (1.19–3.91)	0.0118	2.72 (1.39–5.33)	0.0036
Labour induced with prostaglandin and oxytocin used in labour	6 (6)	5 (3)	3.05 (0.89–10.42)	0.0758	3.92 (1.00–15.33)	0.0494

a Percentage of individuals with complete data.

b Adjusted for woman's age as a continuous linear term, ethnicity, body mass index as a continuous linear term, parity as a continuous linear term, number of previous caesarean deliveries as a continuous linear term, previous uterine surgery, interval between last caesarean section and last menstrual period as a categorical term, and macrosomia.

c Labour either not induced or induced without prostaglandin.

Of the 198 controls (44%) who planned to have a vaginal delivery in their current pregnancy, 40% delivered by caesarean section. The proportion ultimately delivering by caesarean section was similar between those who did and did not have their labour induced and/or received oxytocin in labour (33% [44/132] of control women who laboured without prostaglandin induction or oxytocin in labour; 44% [4/9] of control women who had their labour induced with prostaglandin but did not have oxytocin used in labour; 33% [11/33] of control women who laboured without prostaglandin induction but had oxytocin used in labour; and 20% [1/5] of control women who had their labour induced with prostaglandin and had oxytocin used in labour, *p* = 0.853).

Women with uterine rupture were similar to control women in terms of their Bishop score prior to induction, a measure of the readiness of the cervix for induction (median of 3.5, range 0–8 in women with rupture versus median 4, range 1–7 in control women, *p* = 0.5879). The proportion of women delivering/rupturing before 37 wk amongst those who had prostaglandin induction and/or oxytocin in labour was slightly higher, although not statistically significantly so, in the women with uterine rupture compared to the control women (14% of the women with rupture who had prostaglandin induction and/or oxytocin, 9% of the control women who had prostaglandin induction and/or oxytocin, *p = *0.438). Women with uterine rupture were also similar to control women in terms of the duration of oxytocin use (median 4.8 h, range 0.8–19 in women with rupture versus median 4.8 h, range 0.8–22.5 in control women, *p = *0.7908). While dinoprostone (prostin, propess, prostaglandin E) was the agent used for all the control women induced with prostaglandin, dinoprostone was used in 82% and misoprostol in 18% of the women induced with prostaglandin who had a uterine rupture. Intrauterine death was the indication for induction for all of the women who received misoprostol.

### Characteristics of Uterine Rupture Cases without a Previous Caesarean Delivery

The characteristics of the 20 women who experienced a uterine rupture in the absence of a previous caesarean delivery are shown in Table 6. Compared to a group of population-based control women [14], these women were more likely to be aged 35 y or older (40% versus 20% in control women, uOR 2.74, 95% CI 1.10–6.85) and were more likely to have a parity of three or more (30% versus 9% in control women, uOR 7.62, 95% CI 2.08–27.93). There was also a suggestion that the women who did not have a previous delivery by caesarean section and experienced a uterine rupture were more likely to have an infant with a birthweight of 4,000 g or more (26% versus 12%, uOR 2.69, 95% CI 0.94–7.70), although this was not statistically significant.

**Table 6:** Characteristics of women who had a uterine rupture in the absence of a prior delivery by caesarean section.

Characteristic	*n* (%)^a^ Cases without a Previous Caesarean (*n = *20)
Sociodemographic factors	
**Age (y)**	
Less than 35	12 (60)
35 or older	8 (40)
**Ethnic group**	
White	16 (80)
Non-white	4 (20)
**Socio-economic group**	
Managerial & professional occupations	5 (33)
Other	10 (67)
**Body mass index at booking (kg/m^2^)**	
Less than 25	7 (35)
25–29.9	8 (40)
30 or more	5 (25)
Previous obstetric and medical history	
**Parity**	
0	4 (20)
1–2	10 (50)
3 or more	6 (30)
**Previous uterine surgery**	
No	16 (80)
Yes	4 (20)
**Previous uterine perforation**	
No	19 (95)
Yes	1 (5)
Current pregnancy	
**Twin pregnancy**	
No	20 (100)
Yes	0 (0)
**Placenta praevia**	
No	20 (100)
Yes	0 (0)
**Macrosomia (birthweight 4,000 g or more)**	
No	14 (74)
Yes	5 (26)
**Planned mode of delivery**	
Elective caesarean section	1 (5)
Vaginal	19 (95)
**Induction or oxytocin used in labour in those who planned a vaginal delivery**	
Laboured without prostaglandin induction^b^ or oxytocin in labour	3 (19)
Labour induced with prostaglandin and oxytocin not used in labour	4 (25)
Laboured without prostaglandin induction^b^ but oxytocin in labour	6 (38)
Labour induced with prostaglandin and oxytocin used in labour	3 (19)

a Individuals with complete data.

b Labour either not induced or induced without prostaglandin.

### Outcomes following Uterine Rupture

Two of the 159 women with a uterine rupture died, a case fatality of 1.3%, 95% CI 0.2%–4.5%. 15 (9%) women had a hysterectomy following uterine rupture, ten (6%) women had one or more other organs damaged at rupture or removed during surgery, and 69 (43%) women had other or additional morbidity following their uterine rupture. This group included four women who required ventilation and 62 who received a blood transfusion. 50 (31%) of the woman with a uterine rupture were admitted to critical or high dependency care for a median duration of 2 d (range 1–12).

Outcomes were known for 152 of the infants born to women with a uterine rupture. There were 15 stillbirths (12 antepartum, seven of which occurred prior to uterine rupture in women who were induced following intra-uterine death, and three intrapartum) and ten early neonatal deaths. Excluding the stillbirths that occurred prior to uterine rupture, the perinatal mortality rate was 124 per 1,000 (95% CI 75–189), significantly higher than the national rate of 7.5 per 1,000 (risk ratio [RR] 16.46, 95% CI 10.68–25.39) [15]. Major complications were reported in an additional 19 infants, including nine infants diagnosed with neonatal encephalopathy and six diagnosed with respiratory distress syndrome. A total of 56/137 (41%) of the infants were admitted to a neonatal unit for a median duration of 3 d (range 1–48).

## Discussion

The incidence of complete uterine rupture in the UK as estimated by this study is 1.9 per 10,000 maternities. To our knowledge, our national prospective study using a robust case definition gives one of the first reliable estimates worldwide of the incidence of clinically significant uterine rupture to guide clinical practice, policy, and guidelines. The incidence estimate demonstrates the rarity of clinically significant rupture, and is lower than frequently quoted rupture rates [6].

Consistent with previous caesarean section being the main risk factor in high income countries for uterine rupture [1], our study estimated the incidence to be 11 and 0.3 per 10,000 maternities in women with and without a previous caesarean delivery, respectively.

Amongst women with a previous caesarean section, our study found the risk of uterine rupture was independently increased with increasing number of previous caesarean deliveries; in women with less than a 12-mo interval between their last caesarean section and the start of their current pregnancy; in women with placenta praevia; and in women who planned to have a vaginal delivery compared to those who planned to deliver by elective caesarean section. A higher risk of uterine rupture with labour induction and/or oxytocin use was also apparent. The inclusion of planned mode of delivery in our analysis is the equivalent of intention to treat in randomised controlled trials and fills an important gap in information required to counsel women with a previous caesarean delivery concerning mode of delivery in their next pregnancy [6]. We estimate the incidence of uterine rupture to be 21 per 10,000 maternities in women with a previous caesarean section who planned to have a vaginal delivery in their current pregnancy compared to 3 per 10,000 maternities in women with a previous caesarean section who planned to deliver by elective caesarean section.

### Comparison with Other Studies

A World Health Organization (WHO) systematic review of uterine rupture worldwide, published in 2005, reported an overall median incidence of uterine rupture of 5.3 per 10,000 deliveries based on eight population-based studies identified [1]. Considering only the five population-based studies conducted in a high income country, the incidence was approximately three per 10,000, similar to the overall incidence estimated from our study. One of these five population-based studies was conducted in the UK, and this reported an incidence of two ruptures per 10,000 deliveries (95% CI 1–4, 12 cases) [7], compatible with our overall incidence. However, a more recent larger prospective population-based study in The Netherlands that used very similar methods to our study, reported a higher overall incidence of uterine rupture of 5.9 per 10,000 deliveries (*p*<0.0001), based on 210 cases [16]. This difference may reflect differing rates and patterns of risk factors in the populations. For example, although The Netherlands has a lower caesarean section rate than the UK [17], it appears to have a higher rate of trial of labour after previous caesarean delivery [18]. It also has, for example, unlike the UK [19], a common practice of single rather than double-layer closure of the uterus at caesarean section [16], which has been reported as a risk factor for uterine rupture [20]. We cannot exclude the possibility that the difference is associated with differential case ascertainment. We had no additional sources of data to check our case ascertainment. However, previous studies using UKOSS have suggested high rates of ascertainment. For example, no additional cases were identified through several alternative data sources checked during UKOSS studies of peripartum hysterectomy [14] and acute fatty liver of pregnancy [21]. It is also possible that this observed difference is due to differential reporting of cases according to severity. We specifically excluded women in whom an incidental asymptomatic uterine dehiscence was noted at caesarean section; women with dehiscence were also excluded in the Dutch study, although this may be open to clinical interpretation.

The recent detailed systematic review from the Agency for Healthcare Research and Quality, US Department of Health and Human Services [6] highlights the importance of using an anatomical definition of uterine rupture that specifically excludes asymptomatic dehiscence and notes only four studies using such a definition. Only one of these studies, including a total of just over 6,000 deliveries and 11 uterine ruptures, was population-based. The studies in total included fewer than 48,000 deliveries, compared to the almost 128,000 with a prior caesarean delivery in our study cohort. The review also noted importantly that none of the four studies included information on induction of labour, a factor that could have the potential to considerably influence the incidence rates, as our study shows. This factor is particularly relevant, as the summary results of the review are driven by a single study [22], based in 19 tertiary centres, including 118 ruptures in 33,037 deliveries, which represents a much higher incidence rate than reported in the other included studies.

The WHO systematic review [1] identified only one study giving a rate of uterine rupture in women without a previous caesarean section; a study conducted in a high income country, where the incidence was 0.6 per 10,000 deliveries in women without a previous caesarean section [23], similar to the rate of 0.3 per 10,000 found in our study. For women with a previous caesarean delivery, the WHO review reported a rate of uterine rupture of around 100 per 10,000 based on the available studies in high income countries, much higher than our estimate of 11 per 10,000. This difference is perhaps a reflection of the fact that the WHO estimate was derived predominantly from facility-based studies; the denominator used to estimate the incidence in such studies is likely to be an underestimate of the true denominator of births due to the referral of high risk and emergency cases into the facility from surrounding areas, leading to an overestimate of the incidence. Although we estimated our denominator data, we are confident it is likely to be an accurate reflection of the true denominator: the most recently available national birth data (2009 and 2010 for England and Wales [11] and 2009 for Scotland [12] and Northern Ireland [13]) covering much of the same time period as our study were used to estimate the total number of maternities in the UK over the study period. Also, whilst the proportion of women in the UK with a previous caesarean delivery was estimated from that observed in a study in 2005–2006 [14], this proportion is unlikely to have altered markedly since, as the caesarean section rate in England has not changed substantially between this time and the study period (24.1% in 2005–2006 [24] compared to 24.8% in 2009–2010 [25]).

Other methodological differences may affect observed estimates of the incidence of uterine rupture. A recent population-based study in Australia conducted by retrospective database review of routinely coded data validated by hospital case records reported the incidence of uterine rupture in women with a previous caesarean section delivery as 13 per 10,000 (95% CI 9–18, 37 cases) [26], similar to that found in our study. However, another recent population-based retrospective study in Norway, which also used coded data for case-ascertainment but with limited validation, reported a higher incidence of uterine rupture in women with a previous caesarean section of 50 per 10,000 (94 cases) [27]. This same study reported incidences of 67 and 20 per 10,000 in women with a previous caesarean delivery undergoing a trial of labour or prelabour caesarean section, respectively, higher than that found in our study. An even higher rate of 90 per 10,000 (224 cases) in women attempting a vaginal delivery after a previous caesarean delivery was reported by a recent population-based Swedish study that used unvalidated coded data for case-ascertainment [28]. The use of coded data from routine hospital administrative systems to identify cases of uterine rupture without concurrent chart review can lead to inaccurate case ascertainment [29], which may explain these differences. We therefore suggest that any future studies of this topic should include case validation to ensure robust and comparable results.

Amongst women undergoing a trial of vaginal birth, a recent meta-analysis reported a lower risk of uterine rupture in women with one compared to two prior caesarean sections (pooled OR from five observational studies 0.42, 95% CI 0.29–0.60, *p*<0.0001) [30], compatible with our study findings. A number of studies have also shown a short interdelivery (<18 mo) [31],[32] or interpregnancy (<6 mo) [33],[34] interval is associated with an increased risk of uterine rupture amongst women undergoing a trial of vaginal birth after a previous caesarean delivery. One hypothesis to explain this association is that a short interval leads to incomplete fibrosis of the uterine scar from the previous caesarean delivery, thus increasing the risk of rupture. A study that evaluated incision healing after caesarean section using magnetic resonance imaging reported that at least 6 mo were needed for the zonal anatomy of the uterus to recover [35]. Our findings suggest that women with a previous caesarean section should be advised to wait at least 12 mo before conceiving again.

Agents used to prime the cervix and/or increase uterine contractions such as prostaglandins and oxytocin, can lead to hyperstimulation of the uterus [36],[37] which may weaken scars from previous caesarean sections, increasing the risk of rupture. The recent study by Dekker et al. in Australia [26] is one of the few to have stratified their data by labour induction with or without prostaglandin or oxytocin. Amongst women with one previous caesarean section, compared to those who were not induced and had no oxytocin augmentation, elective caesarean was reported to reduce the odds of rupture while the odds of rupture were increased three- to five-fold in women who had labour induction with or without prostaglandin or oxytocin, six-fold in women who had induction with prostaglandin combined with oxytocin, and 14-fold in women who had augmentation with oxytocin after spontaneous onset of labour. However, these estimates were associated with wide CIs owing to the small number of women with uterine rupture in the study (37 cases), which overlap most of our estimates. A systematic review of labour induction in patients with prior caesarean delivery published in 2005 reported that the use of oxytocin or prostaglandin were associated with a nonsignificant increase in uterine rupture compared to spontaneous labour on the basis of the small number of only fair-quality studies they identified [38]. More recently, the study by Zwart et al. in The Netherlands reported a relative risk of around two in women who had augmentation after spontaneous onset of labour or induction of labour with oxytocin alone or prostaglandin alone compared to spontaneous labour, although they were unable to adjust for any potential confounding factors [16].

### Strengths and Weaknesses of Our Study

A major strength of our study is its population-based national design that reduces the risk of bias associated with facility-based studies. We also used a robust definition of uterine rupture. We were, however, only able to investigate factors that were adequately recorded in the hospital case notes, although this still allowed a comprehensive assessment of the potential risk factors and confounders for uterine rupture and had the advantage that this information was documented prospectively before uterine rupture or delivery, reducing the potential for information bias. We included the phrase “and endangering the life of mother or fetus” in the case definition and our guidance to clinicians, to aid exclusion of women with asymptomatic dehiscence. However, in our analysis we excluded from the cases only women with dehiscence and did not include or exclude any women on the basis of the subjective characteristic of endangered life. We did not exclude women with dehiscence from the control group as these women formed part of the population at risk.

We had no additional sources of data with which to check case ascertainment, so there remains a possibility that we may have under-ascertained cases, or ascertained only more severe cases, although previous studies using this methodology have suggested good case ascertainment. Although we only received data for 75% of the controls requested, we have no evidence of a systematic bias that may affect the validity of our results.

### Conclusions and Policy Implications

Although uterine rupture is associated with significant maternal and perinatal mortality and morbidity, even amongst women with a previous caesarean section planning a vaginal delivery in their current pregnancy, it is rare, occurring in only one of every 500 women. For women with a previous caesarean section, the risk of uterine rupture increases not only with trial of labour but also with the number of previous caesarean deliveries, a short interval since the last caesarean section, and labour induction and/or augmentation. These factors should be considered when counselling and managing the labour of women with a previous caesarean section.

## Abbreviations

aOR: adjusted odds ratio
OR: odds ratio
UKOSS: United Kingdom Obstetric Surveillance System
uOR: unadjusted odds ratio
